# The strength of electron electron correlation in Cs_3_C_60_

**DOI:** 10.1038/srep15240

**Published:** 2015-10-15

**Authors:** L. Baldassarre, A. Perucchi, M. Mitrano, D. Nicoletti, C. Marini, D. Pontiroli, M. Mazzani, M. Aramini, M. Riccó, G. Giovannetti, M. Capone, S. Lupi

**Affiliations:** 1 Center for Life Nano Science@Sapienza, Istituto Italiano di Tecnologia, V.le Regina Elena 291, Rome, Italy; 2 INSTM Udr Trieste-ST and Elettra - Sincrotrone Trieste S.C.p.A. in Area Science park, S.S. 14, km 163,5, Basovizza, Trieste, Italy; 3 Max Planck Institute for the Structure and Dynamics of Matter, 22761 Hamburg, Germany; 4 Center for Free-Electron Laser Science (CFEL), 22761 Hamburg, Germany; 5 CELLS-ALBA, Carretera B.P. 1413, Cerdanyola del Valles, 08290, Spain; 6 Dipartimento di Fisica e Scienze della Terra, Universitá di Parma, Parma, Italy; 7 CNR-IOM-Democritos National Simulation Centre and International School for Advanced Studies (SISSA), Trieste, Italy; 8 CNR-IOM, and Dipartimento di Fisica, Universitá di Roma “La Sapienza”, Rome, Italy

## Abstract

Cs_3_C_60_ is an antiferromagnetic insulator that under pressure (P) becomes metallic and superconducting below *T*_*c*_ = 38 *K*. The superconducting dome present in the *T* − *P* phase diagram close to a magnetic state reminds what found in superconducting cuprates and pnictides, strongly suggesting that superconductivity is not of the conventional Bardeen-Cooper-Schrieffer (BCS) type We investigate the insulator to metal transition induced by pressure in Cs_3_C_60_ by means of infrared spectroscopy supplemented by Dynamical Mean-Field Theory calculations. The insulating compound is driven towards a metallic-like behaviour, while strong correlations survive in the investigated pressure range. The metallization process is accompanied by an enhancement of the Jahn-Teller effect. This shows that electronic correlations are crucial in determining the insulating behaviour at ambient pressure and the bad metallic nature for increasing pressure. On the other hand, the relevance of the Jahn-Teller coupling in the metallic state confirms that phonon coupling survives in the presence of strong correlations.

The allotropic forms of carbon exhibit a wealth of fascinating properties that triggered an intensive research, in particular after the discovery of nanotubes, fullerenes, and graphene^1^,^2^. Indeed, the outstanding mechanical, thermal, electronic, and electrical properties of carbon nanostructures and their functional derivatives, coupled with their chemical robustness, have been the key to the development of a broad range of applications in Physics, Chemistry, and Bio-Medicine^3^. In addition to applications, these materials turned out to be the perfect test bench for studying various physical phenomena, as for instance in fullerides, Insulator-to-Metal transitions (IMT) and Superconductivity: C_60_ is a molecular solid and shows at ambient conditions an f.c.c. structure with a three-fold degenerate t_1*u*_ band^4^. Alkali-intercalated compounds *A*_4_C_60_ are also insulators due to a combined effect of molecular Jahn-Teller (JT) and Mott-Hubbard interactions^5^. *A*_3_C_60_ (*A* = Rb, K) are instead metallic and superconductors with a maximum critical temperature T_*c*_ of 33 K. Superconductivity in *A*_3_C_60_ has been explained in the past through a Bardeen-Cooper-Schrieffer (BCS) phononic mechanism based on the intramolecular *H*_*g*_ Jahn-Teller vibrational modes^6^, which is seemingly supported by a wealth of experimental reports^7^,^8^,^9^.

Even if the JT active phonon modes remain the most likely candidates for pairing, the limitations of a simple BCS phononic mechanism for C_60_ materials start to be evident in compounds with an expanded lattice^10^,^11^,^12^ suggesting a strong role of electronic correlations^13^,^14^,^15^. The synthesis of the insulating, expanded polymorphs of Cs_3_C_60_, as the *A*15 structure, has set a new agenda: Unlike the other members of the A_3_C_60_ family, both f.c.c. and b.c.c. Cs_3_C_60_ are magnetic insulators and become superconducting only under pressure (*P*)^16^, reaching the highest critical temperature in the fullerides, *T*_*c*_ = 38 *K*. The *T* − *P* phase diagram of Cs_3_C_60_ shows the emergence of superconductivity from an insulating antiferromagnetic parent state, resembling what found in other high-temperature superconducting families^17^,^18^,^19^ and calls for a deeper understanding of the role of electron-electron interactions in *A*_3_C_60_ compounds^20^,^21^, and in particular in Cs_3_C_60_^22^.

In this work we have combined novel infrared experimental data with theoretical calculations to shed more light on the strength of electron correlation and on the evolution of the Jahn-Teller interaction in *A*15 Cs_3_C_60_ under pressure. Infrared spectroscopy is a powerful technique to study the low-energy electrodynamics of solids as it yields information on both the electronic and vibrational degrees of freedom. By measuring the optical conductivity, the pressure-driven metallization process can be traced by the emergence of a Drude term in the spectra. On the other hand, a splitting of the phonon lines indicates a symmetry reduction, possibly associated with a Jahn-Teller distortion.

In this paper we investigate the low-energy electrodynamics and the insulator to metal transition of Cs_3_C_60_ under pressure at room temperature. We find that a strongly correlated bad metallic state is established at rather low pressures in concomitance with the Jahn-Teller distortion which maintains and stabilizes in the metallic phase. This result suggests that in the proximity of the Mott transition may enhance the electron-phonon interaction leading to the relatively high superconducting critical temperature in the *A*15 structure.

## Results

### Infrared Data

**Figure 1:**
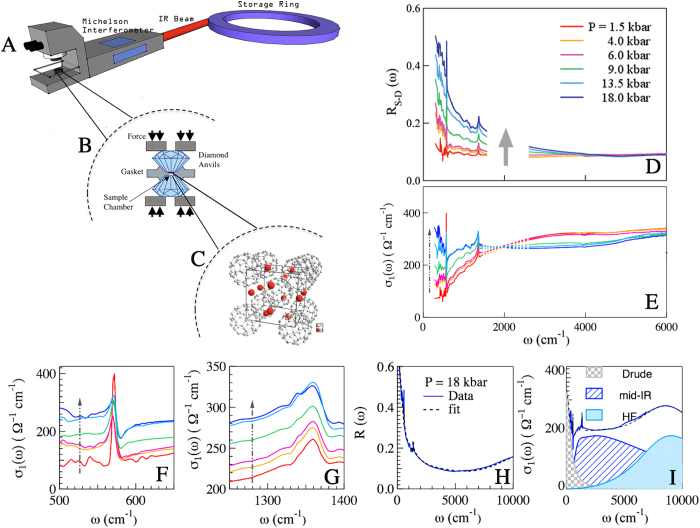
(**A**) Sketch of the experimental end station of SISSI beam line at ELETTRA Storage Ring. A Michelson interferometer from Bruker is coupled to an IR Hyperion 2000 microscope able to measure both the infrared spectra and, in the same experimental condition, the ruby fluorescence. (**B**) Cartoon of the Diamon Anvil Cell (DAC). A Cs_3_C_60_ pellet is placed in the DAC sample chamber creating a clean interface with the upper diamond culet; (**C**) Crystal structure of A-15 Cs_3_C_60_; (**D**) Reflectivity at the sample-diamond interface at a number of pressures indicated in Figure. The arrow indicates the direction of increasing pressure. Data are not shown between 1700–2600 cm^−1^ due to not perfect compensation of the diamond phonon absorptions in this spectral region. (**E**) The optical conductivity is shown in the same frequency range and at the same pressures. The dashed area corresponds to the frequency range where diamond absorption occurs; *σ*_1_(*ω*) is shown in (**F**,**G**) over a reduced frequency range to better highlight the evolution of the phonon modes with pressure; In (**H**,**I**) are reported the experimental reflectivity and the optical conductivity obtained by Kramers-Kronig transformation with the fitting curves resulting from simultaneous fitting of *R*_*S*−*D*_ and *σ*_1_(*ω*) with a Drude-Lorenz model.

At ambient conditions the reflectivity at the sample-diamond interface *R*_*S*−*D*_ is almost flat, and shows the infrared signatures of the *T*_1*u*_ vibrational modes at about 570 and 1370 cm^−1^. For increasing values of the applied pressure (indicated in Fig. 1 by the grey arrow), *R*_*S*−*D*_ raises towards low-frequency, suggesting the onset of a carrier-delocalisation process. In Fig. 1E are reported the optical conductivity curves *σ*_1_(*ω*) achieved by Kramers-Kronig transformation from *R*_*S*−*D*_ data (see the Method section) up to 6000 cm^−1^. Already at 4.0 and 6.0 kbar the conductivity level increases towards low frequency through a transfer of spectral weight from above to below an isosbestic point around 2000 cm^−1^. The insulator to metal transition occurs around 13 kbar where a Drude term clearly shows up in the spectra and *σ*_1_(*ω*) monotonically increases for *ω* → 0. Noteworthy, in the metallic phase of Cs_3_C_60_ the absolute value of *σ*_1_(*ω*) is that of a poor metal as also observed in Rb_3_C_60_, K_3_C_60_^23^ and in Na_2_CsC_60_^24^.

At the lowest pressure the phonon mode at 570 cm^−1^ is symmetric (see Fig. 1F), as found also in ref. 25. When the system at high pressure becomes metallic, this phonon mode couples with the electronic continuum and shows the signature of a Fano-like distortion, similarly to what found in the metallic *A*_3_C_60_ (*A* = Rb, K) compounds. The phonon mode centered at 1370 cm^−1^ (Fig. 1G) shows instead a splitting in three peaks, that becomes more evident at the highest measured pressure. Let us observe that the splitting of this phonon is in excellent agreement with what shown in ref. 25, also perfectly confirming the calculations performed in ref. 26.

## Discussion

### Electronic correlation

To assess the importance and the strength of the Coulomb repulsion *U* in determining the ground state of Cs_3_C_60_ we can compare the experimental results with theoretical calculations.

**Figure 2:**
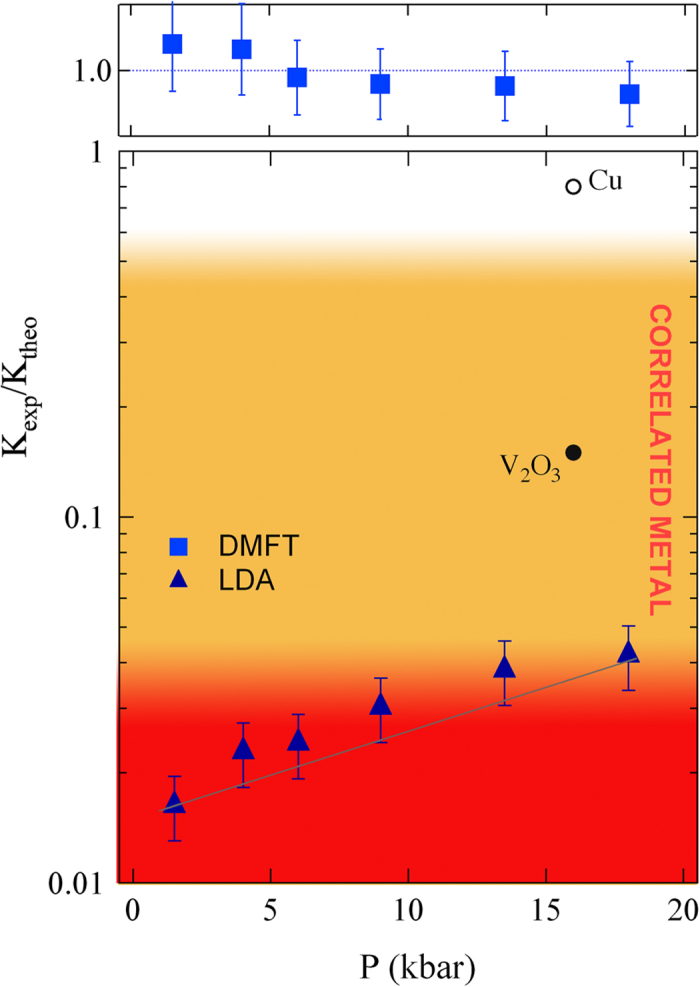
Evolution with pressure of the degree of correlation. The ratio of the experimental and the “mean-field” theoretical kinetic energy *K*_*exp*_/*K*_*LDA*_ ratio (triangles) is compared with data from ref. 28 on V_2_O_3_ and Cu. *K*_*exp*_/*K*_*LDA*_ increases with increasing pressure indicating a smooth transition from a Mott insulator to a correlated metal. On the other hand, *K*_*exp*_/*K*_*DMFT*_ (squares) is nearly constant at 1. This indicates that DMFT is taking correctly into account the electron-electron correlation, capturing the pressure-driven appearance of the quasi-particle peak.

The ratio between the experimental kinetic energy (*K*_*exp*_) and that obtained via band structure “mean-field” calculations (*K*_*LDA*_) can be used to determine the “degree of correlation” of a material. Such a ratio spans between 0 (characteristic of a Mott insulator) to 1, as in the case of conventional metals^27^,^28^,^29^,^30^. The *K*_*exp*_/*K*_*LDA*_ ratio is shown in Fig. 2 for Cs_3_C_60_. *K*_*exp*_ was obtained from the integral of *σ*_1_(*ω*) for a cut-off frequency of 900 cm^−1^, which captures pratically all the Drude spectral-weigth, as can be seen in the Drude-Lorenz fit shown in Fig. 1D. The error-bars in Fig. 2 take into account an indetermination of ±100 cm^−1^ in choosing the cutoff energy for the Drude term. *K*_*LDA*_ are instead calculated through Density-Functional Theory (DFT) using the crystal structure relative to different pressure values (see Methods). *K*_*exp*_/*K*_*LDA*_ values are below 0.1, placing Cs_3_C_60_ at the verge of the Mott transition^27^. With increasing pressure Kexp/KLDA raises, mirroring the increase of spectral weight at the Fermi energy, i.e. representing the emergence of a quasi-particle peak in the density of states. The Drude peak that shows up in Cs_3_C_60_ above 13 kbar does not resemble that measured in the metallic phases of K_3_C_60_ or Rb_3_C_60_^31^. Here, a broad Drude term can be found, superimposed to the HOMO-LUMO excitations at about 1 eV. Instead in Cs_3_C_60_, a relatively small Drude term is present, whereas a broad absorption band is still visible slightly below 2000 cm^−1^.

**Figure 3:**
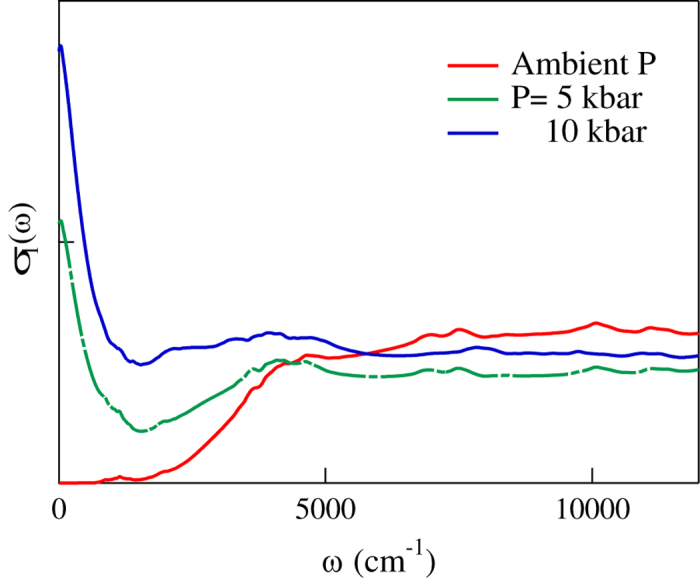
Optical conductivity curves obtained by DMFT calculations at room temperature, at ambient pressure and at 5 and 10 kbar.

To further understand the importance of electron-electron correlation on Cs_3_C_60_, we compare the experimental results with calculations combining DFT with Dynamical Mean-Field Theory (DMFT). The impurity model is solved at 300 K by finite-temperature exact diagonalization^32^. From DMFT we have computed *K*_*exp*_/*K*_*DMFT*_ that turns out to be (Fig. 2) nearly constant (and equal to 1), for increasing pressure. This highlights that DMFT correctly captures the pressure-driven appearance of the quasi-particle peak and its effect on the kinetic energy. (see Fig. 2). The corresponding theoretical optical conductivity curves are reported in Fig. 3 where it is possible to follow the partial closure of the gap and at the same time the growth of the Drude term by increasing pressure. The theoretical result describes the evolution of *σ*_1_(*ω*) with pressure accurately, corroborating that electron-electron correlation plays a major role in the physics of Cs_3_C_60_ and mainly determines the Insulator-to-Metal transition. Noteworthy, both theoretical and experimental curves show the coexistence of the Drude term with a mid-infrared absorption band, as an infrared signature of strong correlation^33^. This also occurs at the pressure values where superconductivity is found at low temperature, confirming the belief that the superconductivity emerges from a strongly correlated metallic phase^20^.

### Phonon modes

Besides the strong electron-electron interactions, the molecular Jahn-Teller effect plays a pivotal role in the physics of fullerides. The coupling with the Jahn-Teller active H_1*g*_ modes is the largest contribution to the electron-phonon coupling, and it is widely believed to be responsible of the superconducting pairing. In particular, it has been demonstrated that a Jahn-Teller coupling can survive and even benefit from the strong correlations identified in the present study^14^.

Recently some authors^25^ have discussed the fingerprints of the molecular Jahn-Teller effect in the optical spectra of Cs_3_C_60_ at ambient pressure. By lowering the temperature below 300 K, a splitting of this phonon mode has been detected, hinting to the presence of a dynamical Jahn-Teller distortion. While at room temperature and above, the thermal expansion results in a weaker crystal field that does not select a preferred direction of the distorted molecule, by decreasing *T* the population of two possible solid-state conformers starts to differ and a clear splitting in the phonon mode at 1370 cm^−1^ is found.

In our study a shoulder in the 1370 cm^−1^ phonon is visible already at the lowest measured pressure, however the splitting becomes more evident for increasing pressure, even at room-*T*, where instead at ambient pressure, it was argued^25^ that no preferred direction was chosen by the distorted molecule. A preferred direction seems to be present instead at high-pressure, likely due to a modification of the local crystal field with pressure. A similar behaviour has been recently discussed on Rb_x_Cs_3-x_C_60_, where the interfulleride distance is controlled via chemical substitution rather than by external pressure^34^.

Therefore, our data show that pressure can drive the system metallic while maintaining (and stabilizing) a dynamical Jahn-Teller distortion. These results confirm that the Jahn-Teller effect is not a main force in the stabilization of the insulating behavior, which is indeed solely due to the Mott localization of carriers. Interestingly, the present data show that the Jahn-Teller coupling is more effective when the system becomes metallic, either by increasing the pressure or reducing the temperature, a result fully compatible with the scenario in which a Jahn-Teller coupling leads to superconductivity in the strongly correlated metal close to the Mott transition^20^.

## Conclusions

In conclusion, pressures in the kbar range are sufficient to drive Cs_3_C_60_ in a metallic state, while signatures of strong electron correlation persist up to the highest measured pressure. Cs_3_C_60_ is thus found to be at the verge of a Mott-Hubbard Insulator-to-Metal transition: by applying an external pressure a carrier-delocalization is induced corresponding to a poor-metallic state where dynamical Jahn-Teller distortions are maintained and stabilized.

The comparisons with DMFT theoretical calculations support the claim that electron- electron correlation is the sole responsible for the insulating state at ambient conditions and can alone explain the low-energy Cs_3_C_60_ evolution across the IMT. Noteworthy, strong correlation persists at high pressure and therefore it has to be considered in order to reach a full understanding of superconductivity in Cs_3_C_60_. On the other hand, the relevance of the Jahn-Teller coupling in the metallic state confirms the theoretical scenario in which the phonon coupling survives in the presence of strong correlations.

## Methods

### Sample preparation and characterization

A15-rich Cs_3_C_60_ sample was obtained via a solvent-mediated synthesis route. Stoichiometric amount of Cs metal (Aldrich, >99.5% purity) and C_60_ powder (MER >99.9%) were put in a Pyrex vial in a controlled atmosphere (Ar glove-box, <0.1 ppm O_2_ and H_2_0), having particular care to avoid the direct contact between the reagents in this stage. The vial was then evacuated (P < 10^4^ mbar) and placed in a methanol bath at −60 degrees Celsius and previously degassed anhydrous methylamine (Aldrich >98% purity) was condensed under continuous stirring. At this step the solution became dark-red, due to the dissolution of the alkali metal in the solvent. Hence, the vessel was sealed and slowly heated at T = 50 degrees Celsius and let react for one night under stirring. After reaction took place the color of the suspension became dark brown. Methylamine was slowly evaporated at −5 degrees Celsius and the dry product was collected in glove box, then it was pelletized and further treated in dynamic vacuum for 20 h at 200 degrees Celsius. Quantitative Phase Analysis (QPA) of the product was performed by synchrotron radiation powder diffraction, and indicated a phase fraction of respectively 74(1)% A15, 14(1)% fcc and 12(1)% bco.

### Infrared Spectroscopy under pressure

High pressure measurements were performed with a screw-driven opposing plate Diamond-Anvil-Cell. An Al gasket was chosen to span over the desired pressure range (0–20 kbar) in a reproducible and cyclic way. A hole of about 300 *μ*m diameter was drilled in the pre-indented gasket. A small amount of Cs_3_C_60_ sample was pressed between the diamond anvils to form a pellet with a thickness of about tens of microns^35^,^36^. This thickness yields zero transmission, allowing for reflectivity measurements. The pellet was then loaded in the cell with CsI as pressure medium, taking great care of obtaining a clean diamond-sample interface. As these samples are strongly air-sensitive the whole procedure was done in a glove box and the pressure cell was carefully kept closed for the experiment. The pressure was measured *in*-*situ* by the standard ruby fluorescence technique^37^ with a procedure described elsewhere^38^. Infrared reflectivity measurements were performed with the aid of the Hyperion 2000 Bruker microscope coupled to an IFv66/s Michelson interferometer, exploiting the high brilliance of synchrotron radiation at the infrared beam line SISSI of ELETTRA storage ring^39^. Reflectivity at the sample diamond interface *R*_*S*−*D*_ was measured with 1 cm^−1^ resolution between 250 ÷ 15000 cm^−1^. The real part of the optical conductivity *σ*_1_(*ω*) was then extracted via Kramers-Kronig (KK) transformations taking care of the sample diamond interface^38^,^40^. Simultaneaous fitting of *R*_*S*−*D*_ and *σ*_1_(*ω*) with a Drude-Lorenz model is performed to make sure that the KK procedure has been performed correctly.

### Dynamical Mean Field Theory calculations

Our theoretical data are based on the combination of density functional theory (DFT) with Dynamical Mean-Field Theory (DMFT). The DFT band structure of Cs_3_C_60_ has been calculated using the Perdew-Burke-Ernzerhof recipe for the genealized gradient approximation by means of the Quantum Espresso package^41^ employing a grid of 6 × 6 × 6 k-points. The cutoff energies for wavefunctions and charge densities were set to 45 Ry and 450 Ry, respectively. The calculations are based on the A15 structure of Cs_3_C_60_ with Pm3n symmetry with b.c.c. anion packing^23^ at ambient pressure without performing structural relaxations, and the pressure dependence is deduced from the experimental data for the lattice spacings. A tight-binding representation of the band structure is built using Wannier90^42^ to compute the maximally localized Wannier orbitals restricting to the bands originated from the t_1*u*_ LUMO.

We combine the DFT band structure with the on-site interactions acting on the three-fold degenerate t_1*u*_ manifold. We considered the large Hubbard *U* estimated in ref. 43, and we included an attraction term which results from the Jahn-Teller electron-phonon interaction, even if significantly reduced by the Hund’s rule coupling, which involves exactly the same operators^5^. As we did not consider structural relaxations, we do not account for the static Jahn-Teller distortions, while we account for the dynamical Jahn-Teller interaction and its effects of the electronic properties. Indeed the two interaction terms have been shown to reproduce the low-temperature phase-diagram of Cs_3_C_60_ within model calculations.

Here, we chose the value of the attractive interaction in order to reproduce the spectral gap of the insulating compound at ambient pressure. The value used is *J* = 0.07 *eV*. The DMFT calculations employ an exact diagonalization solver at finite temperature in the implementation of 32 4 impurity levels per orbital are included for a total number of 15 levels including the impurity site, and the full orbital rotational symmetry is implemented to reduce the Hilbert space. The optical conductivity is computed from the single-particle Green’s functions including the vertex functions obtained by differentiation of the DFT band structure with respect to momentum.

## Additional Information

**How to cite this article**: Baldassarre, L. *et al.* The strength of electron electron correlation in Cs_3_C_60_. *Sci. Rep.* **5**, 15240; doi: 10.1038/srep15240 (2015).
